# Typical CT Pattern in Rheumatoid Arthritis: Exuberant Honeycombing

**DOI:** 10.5334/jbsr.4109

**Published:** 2025-10-31

**Authors:** Cédric Descatoire, Pascale Bohy

**Affiliations:** 1 Hôpital Universitaire de Bruxelles (HUB), Belgium

**Keywords:** HRCT, rheumatoid arthritis, exuberant honeycombing

## Abstract

*Teaching point:* Exuberant honeycombing on high-resolution computed tomography of the chest is a specific sign of connective tissue disease-associated interstitial lung disease with a usual interstitial pneumonia pattern and contributes to differentiate it from idiopathic pulmonary fibrosis.

## Case History

A 58-year-old North African female, living in Belgium since childhood, known with a 9-year history of rheumatoid arthritis (RA) treated with methotrexate and Medrol, was referred for imaging studies to assess respiratory restriction, reduced diffusing capacity for carbon monoxide and peripheral crackles.

**Figure 1:**
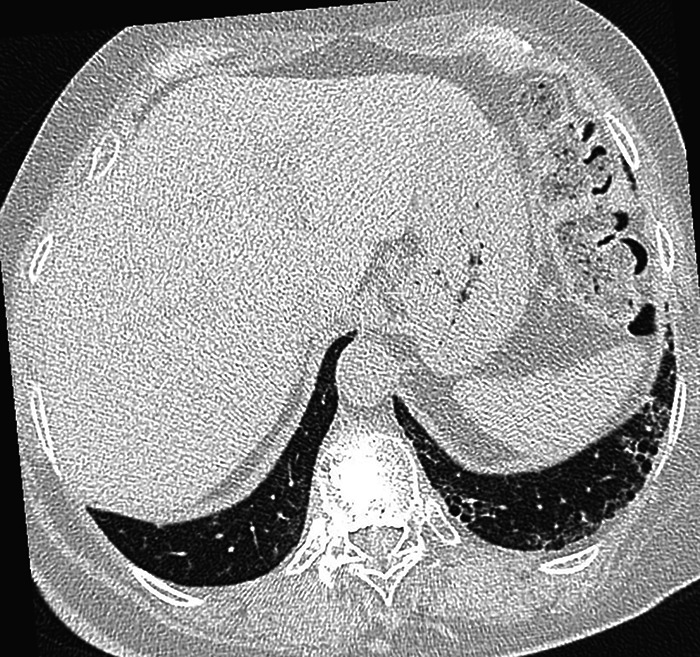
Axial initial CT image of lungs showing bi-basal cystic images, especially on the left and reminiscent of honeycomb, suggestive of early pulmonary fibrosis.

A chest radiograph, performed six weeks before methotrexate initiation, was unremarkable. A chest CT performed in February 2015 to rule out methotrexate toxicity and early interstitial pathology showed bilateral basal cystic changes, particularly on the left, reminiscent of honeycomb, suggestive of early pulmonary fibrosis (Figure 1).

**Figure 2:**
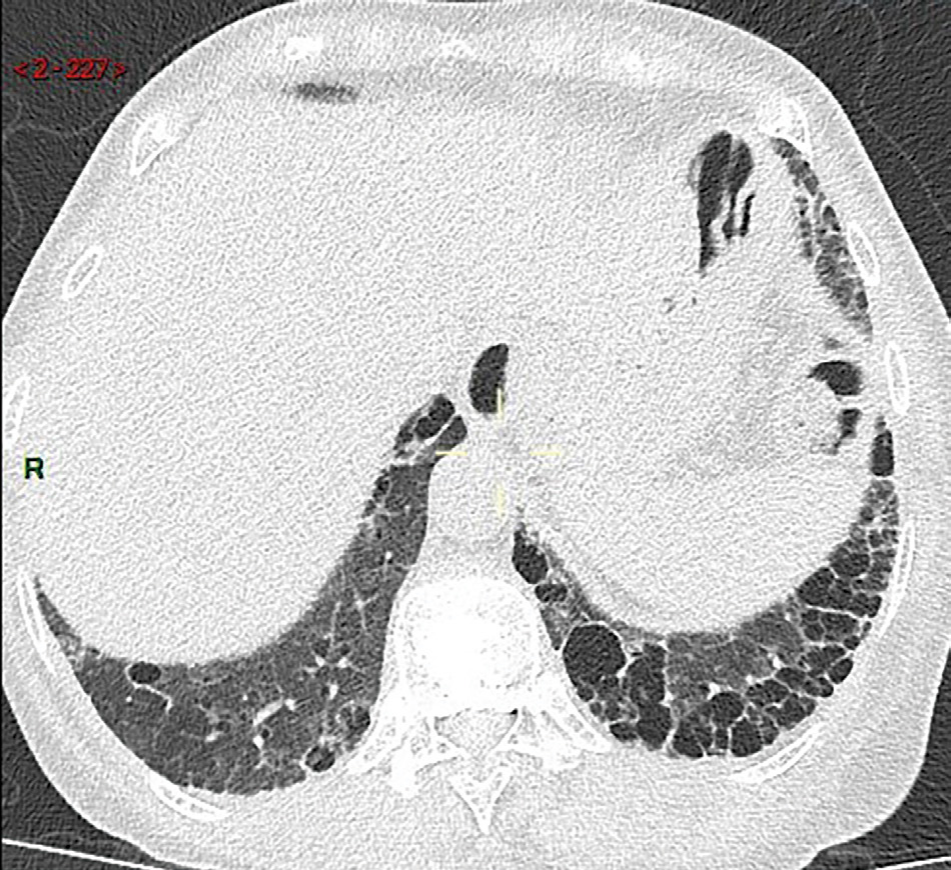
Axial follow-up CT image of lungs showing exuberant and macrocystic honeycomb at the periphery of the pulmonary bases, increasing compared to previous exam.

**Figure 3:**
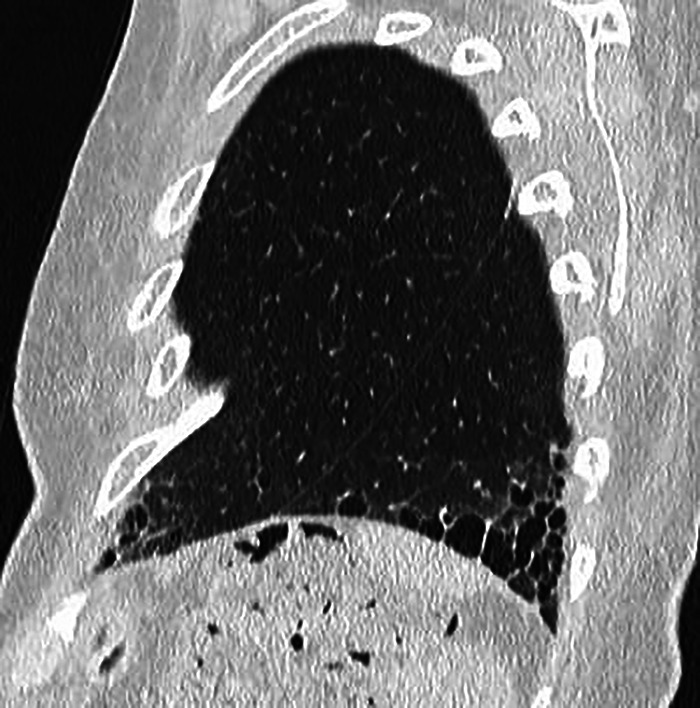
Sagittal follow-up CT image of lungs showing exuberant and macrocystic honeycomb at the periphery of the pulmonary bases, increasing compared to previous exam.

A CT performed 9 years later (May 2024) demonstrated marked progression, with extensive honeycomb-like cysts predominantly at the bases. According to the Fleischner Society, exuberant honeycombing is defined as confluent macrocystic changes involving more than 70% of the fibrotic lung areas at the periphery of the pulmonary bases. This radiological feature is considered a strong indicator of connective tissue disease-interstitial lung disease (CTD-ILD) rather than idiopathic pulmonary fibrosis (IPF) (Figure 2 and Figure 3).

## Comment

RA-ILD is the most frequent extra-articular manifestation of RA and contributes significantly to morbidity and mortality. High-resolution computed tomography (HRCT) typically shows two main patterns: Non-specific interstitial pneumonia (NSIP) and usual interstitial pneumonia (UIP). UIP is the most frequent, reported in about 60% of RA-ILD cases, and is associated with a poorer prognosis, characterized by progressive fibrosis and reduced survival compared to non-UIP patterns. In typical cases, HRCT findings are considered sufficient for diagnosis, and histological confirmation is not required [1].

In this patient, the long-term progression, basal predominance, and extension of honeycombing argued against drug toxicity and confirmed RA-ILD with a UIP pattern. Recognizing exuberant honeycombing is important because it narrows the differential diagnosis, indicates a poor prognosis, and highlights the need for close multidisciplinary follow-up.

## Conclusion

This case illustrates the progressive course of RA-ILD with exuberant honeycombing, a CT sign strongly suggestive of CTD-ILD with a UIP-pattern.
